# Prospective strategies to delay the evolution of anti-malarial drug resistance: weighing the uncertainty

**DOI:** 10.1186/1475-2875-9-217

**Published:** 2010-07-23

**Authors:** David L Smith, Eili Y Klein, F Ellis McKenzie, Ramanan Laxminarayan

**Affiliations:** 1 Emerging Pathogens Institute and Department of Biology, University of Florida, Bartram-Carr Hall, Room 614, P.O Box 118525, Gainesville FL 32611, USA; 2 Resources for the Future, 1616 P Street NW, Washington, DC, USA; 3 Department of Ecology & Evolutionary Biology, Princeton University, 106A Guyot Hall, Princeton, NJ 08544, USA; 4 Princeton Environmental Institute, Princeton University, 129 Guyot Hall, Princeton, NJ 08544, USA; 5 Fogarty International Center, National Institutes of Health, 31 Center Drive - MSC 2220, Bethesda, MD 20892, USA

## Abstract

**Background:**

The evolution of drug resistance in malaria parasites highlights a need to identify and evaluate strategies that could extend the useful therapeutic life of anti-malarial drugs. Such strategies are deployed to best effect before resistance has emerged, under conditions of great uncertainty.

**Methods:**

Here, the emergence and spread of resistance was modelled using a hybrid framework to evaluate prospective strategies, estimate the time to drug failure, and weigh uncertainty. The waiting time to appearance was estimated as the product of low mutation rates, drug pressure, and parasite population sizes during treatment. Stochastic persistence and the waiting time to establishment were simulated as an evolving branching process. The subsequent spread of resistance was simulated in simple epidemiological models.

**Results:**

Using this framework, the waiting time to the failure of artemisinin combination therapy (ACT) for malaria was estimated, and a policy of multiple first-line therapies (MFTs) was evaluated. The models quantify the effects of reducing drug pressure in delaying appearance, reducing the chances of establishment, and slowing spread. By using two first-line therapies in a population, it is possible to reduce drug pressure while still treating the full complement of cases.

**Conclusions:**

At a global scale, because of uncertainty about the time to the emergence of ACT resistance, there was a strong case for MFTs to guard against early failure. Our study recommends developing operationally feasible strategies for implementing MFTs, such as distributing different ACTs at the clinic and for home-based care, or formulating different ACTs for children and adults.

## Background

*Plasmodium falciparum*, which causes malaria, is the most important parasite species that infects humans with approximately 2.37 billion people at risk [1,2]. Prompt effective drug treatment can reduce the risk of mortality for those with clinical infections, and is a key component of malaria elimination and eradication plans both past and present [3]. Diminished therapeutic efficacy due to the evolution of resistance to previous first-line drugs, however, contributed to the failure of initial eradication efforts and resulted in increases in infection and mortality [4]. Drug stewardship, combination therapies, and other policies have been advocated to slow the evolution of resistance to anti-malarial drugs, however to be effective, a policy must delay emergence or at least slow the spread of resistant parasites. Combination of drugs, such as artemisinin combination therapy (ACT), delay emergence by eliminating resistant mutants except those that carry two different mutations. Since each drug can eliminate mutants that are resistant to the other component, mutations to both components must arise in the same parasite. Combination therapies thus delay the time until a viable resistant parasite appears [5]. Alternative strategies call for reducing selection for the spread of resistance by using drugs more prudently or by rationing drugs [6]. Such strategies assume that the rate of spread increases in proportion to the rate of drug use in a population. Multiple first-line therapies (MFTs) on the other hand, reduce pressure on each drug, defined as the proportion of clinical episodes that are treated, and slow spread while avoiding the ethical problem of leaving some patients untreated [7,8].

**Figure 1:**
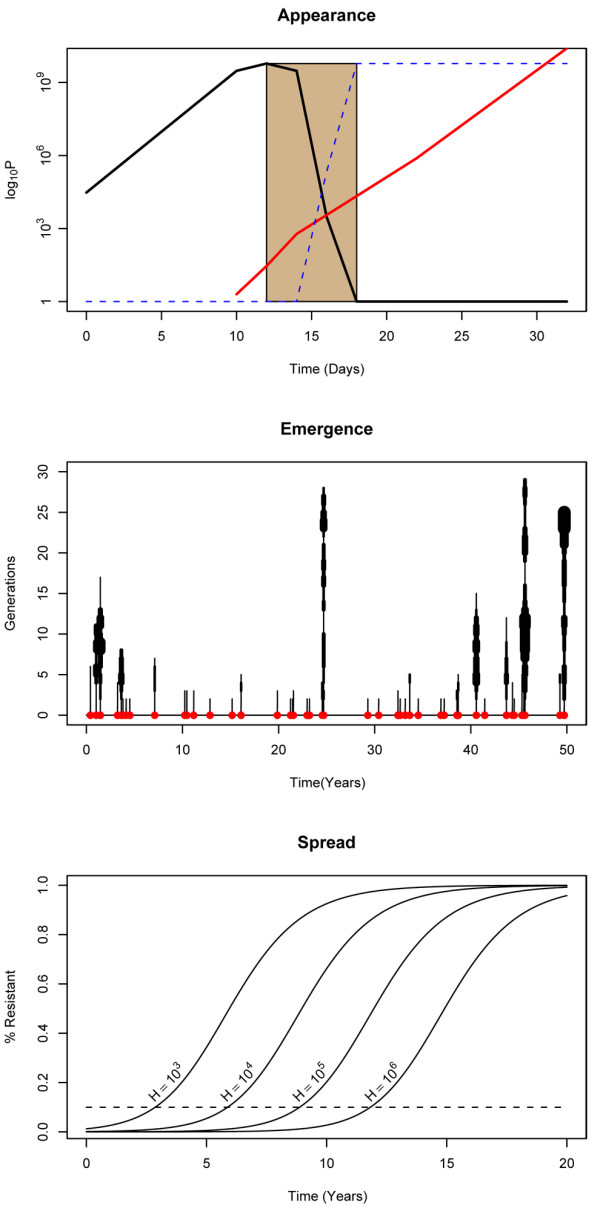
**The evolution of anti-malarial drug resistance**. The evolution of anti-malarial drug resistance is shown schematically in three steps: *Top*) *Appearance *involves *de novo *mutation and within-host selection by drugs (brown). Sensitive parasites (black) initially outnumber resistant mutants (red), but after treatment, the proportion of parasites that are resistant (blue) increases, and so too does the likelihood of transmitting a resistant parasite. *Middle*) Emergence involves sporadic appearance (red) and stochastic establishment. *Establishment *involves generating "progeny" (infected humans) and becoming common enough to avoid stochastic fadeout. In the generations after appearance, the number of progeny fluctuates. This was simulated as an evolving branching process and illustrated with vertical lines whose thickness corresponds to the number of progeny in that generation. In the figure, the last one successfully established, defined as a point when 100 "offspring" existed with positive fitness, and spread was considered virtually certain. *Bottom*) After emergence, the *spread *of resistance was simulated in simple epidemiological models. Starting from 100 individuals who carried resistant parasites, the time to failure (10%, dashed line) depended strongly on the human population size (H).

While combination therapies delay emergence and MFTs slow spread, this simple analysis ignores the other effects of reducing drug pressure. Emergence itself is a complicated process that begins with random mutations and drug treatment, followed by transmission and stochastic persistence until a mutation(s) conferring resistance has become firmly established in the population. Mutations that confer drug resistance can arise any time a parasite replicates, but drugs must reduce the density of drug-sensitive parasites to allow resistant parasites to thrive within a human and have a reasonable chance of being transmitted to another human (Figure 1, top) [9]. Appearance of resistance is the result of within-host selection of a new mutant, not just mutation, so the rate of appearance is proportional to drug pressure.

*De novo *resistant mutants may fail to become established, as the parasite is present in only a few hosts, and thus its persistence is a matter of chance [10,11] (Figure 1, middle). The fitness advantage for drug-resistant parasites under some level of drug pressure is partially offset by a fitness disadvantage, or a biological cost of resistance, if drugs are absent [12-15]. The biological cost will tend to be highest when resistance first appears and will reduce the probability of establishment because mutations that lower fitness tend to die out [12,13]. Therefore, reducing drug pressure would also reduce the chances of establishment by increasing the effect of a high initial cost of resistance.

Thus, reducing drug pressure impacts the evolution of resistance in three ways: it delays its appearance, reduces the likelihood of its establishment, and slows its spread (Figure 1, bottom). However, the evolution of drug resistance involves complex nonlinear processes, and thus substantial uncertainty exists about the relative impact of deploying MFTs because of unknown mutation rates, poor information about drug pressure, and a poorly defined relationship between drug pressure and fitness. Weighing uncertainty requires a quantitative approach using mathematical models. In this paper, the effectiveness of deploying multiple ACTs to manage resistance was evaluated using a hybrid modelling approach that weighs all three potential benefits of deploying MFTs.

## Methods

Most new first-line anti-malarial drugs as well as most anti-malarial drugs in the development pipeline contain an artemisinin-class drug. To extend their useful therapeutic lives, artemisinins are paired with a partner drug and given as combination therapies. While several different ACTs are already available on the market, artemisinin monotherapy is still readily available in many places. Cross-resistance among all the artemisinin derivatives is expected, and the evolution of resistance to a partner drug should be comparatively easy for a parasite with artemisinin resistance, so the loss of one ACT would probably lead to the rapid loss of all ACTs. Because new non-artemisinin anti-malarial drugs will likely not be available for a decade or more, preserving artemisinin is crucial. A significant threat to artemisinin comes from the evolution of resistance to the partner drugs, whose pharmacokinetic half-lives are generally significantly longer than the artemisin-class drugs they are paired with. This has led to concerns about selection for partner drug resistance after the artemisinin concentrations have waned [5,16]. MFTs would provide one way for the partner drugs to protect each other and, by extension, artemisinin. To examine the full benefit of MFTs, a quantitative approach to assessing the waiting time to ACT failure was developed.

### Appearance

Appearance, defined as an infection with *de novo *mutants that are frequent enough within a single host to be transmitted to another host, requires a resistant mutant and treatment (Figure 1, top). The probability that at least one resistant mutant is present when chemotherapy begins is determined by the probability of spontaneously generating a resistant mutant per cell division, *m*, and the parasite population size at the time of treatment, *P*. The probability of appearance is thus approximately *mP *(assuming it is small, i.e. *mP < 0.01*).

The time to appearance, *T_a_*, in a human population of size *H *depends on the distribution of parasite population sizes at the start of treatment, Y(*P*). *De novo *resistance appears with some low probability each time a person is treated: *∫ mP *Y*(P) dP*. The waiting time to appearance also depends on the number of people who are treated which depends on per-capita clinical incidence *L*, the human population size, *H*, and drug pressure, or the proportion of clinical episodes that are treated, *p; *the number of clinical episodes that are treated per year is thus *pLH*. The waiting time to appearance is exponentially distributed with mean:.

**Figure 2:**
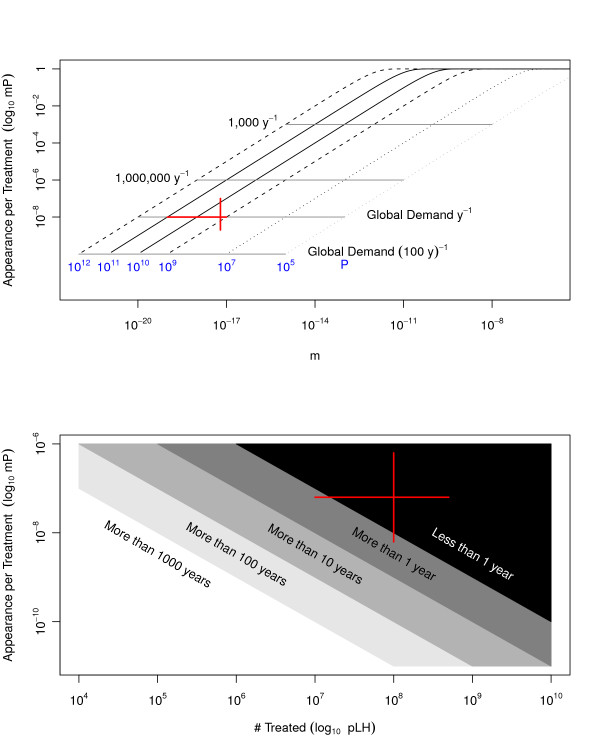
**The waiting time to appearance**. *Top*) The waiting time to appearance in a treated individual is plotted against the mutation rate for a range of parasite densities. Horizontal lines mark the point where exactly one appearance is expected in treated populations of different size. *Bottom*) Colors encode categorical descriptions of the time to appearance for parameters that span the relevant range for the number of people treated in one year and the probability of appearance, per treatment. In both panels, the red lines are centered on a mutation rate of *10 *^*-17.2 *^(i.e., two independent mutations at the rate for mutations that confer SP resistance), parasite densities of *10 *^*10*^, and 1 billion treatment courses per decade. The lines span parasite densities from *10 *^*9 *^to *10 *^*11 *^and global demand from 100 million to 5 billion.

Given an estimate of the mutation rate and the distribution of parasite population sizes, it is possible to compute the expected number of people who could be treated before resistance would appear once (Figure 2). Time to appearance is inversely proportional to human population size and drug pressure: if the number of people taking a drug is cut in half, then the expected waiting time to emergence is twice as long. Human population size is not like the other parameters in that the choice of *H*, up to the global population at risk [17], defines the scale of the problem being considered.

### Establishment

After appearance, a parasite can only become established by spreading to other hosts. Parasite fitness is measured in terms of the replacement number, the expected number of human hosts that would be infected for each human host infected. The fitness of a resistant phenotype is determined by its biological cost and by the advantage conferred from drug pressure. If a parasite cannot replace itself, it will never become established, though parasite fitness may be too low for it to become established initially. The fitness of a resistant phenotype will tend to increase if they persist, as they can acquire compensatory mutations to overcome the biological cost of resistance. Both persistence and the acquisition of compensatory mutations are chance events, though the compensatory mutations increase the likelihood of persistence.

Establishment of resistance was considered in a population where malaria is endemic, and where on average parasites replace themselves approximately once. Parasites with *de novo *resistance are rare in a population when they first appear, by definition, and when replacement numbers are close to one, they frequently fail to establish.

The probability of establishment, *Q*, and the number of parasite generations required to establish, *G*, were estimated by simulating an evolving branching process [10,11,18]. A branching process does not keep track of when the progeny were produced, but rather the number of offspring in each generation that has elapsed (Figure 1, middle). The number of "offspring" (i.e., new human infections) was drawn from a negative binomial distribution to account for heterogeneous biting [19,20]. The offspring evolved by inheriting a slightly different fitness than their parents. The branching process was iterated until no parasite offspring remained (a failure) or there were at least 100 offspring with replacement numbers greater than one (a success) (Additional file 1). It was repeated *n *times until there were *s *successes, and *Q ≈ (s-1)/(n + s-1)*. Assuming that the time required to complete a parasite generation was *T_g _*(i.e. the average time from one human infection to another, at least one month and generally approximately two months), the waiting time to emerge was

Failure to establish has the effect of delaying emergence as much as requiring an additional mutation that occurs with frequency *Q*.

**Figure 3:**
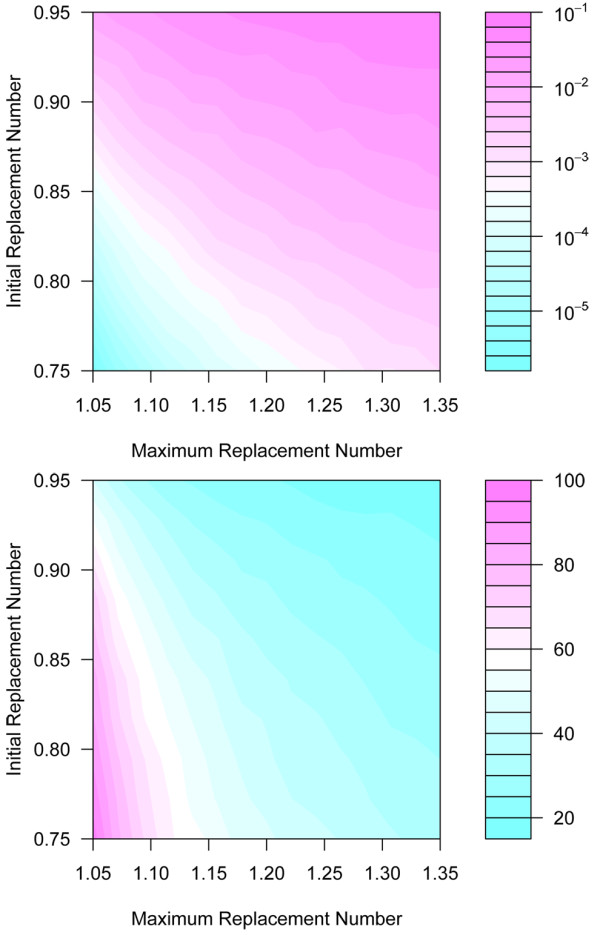
**Summary statistics from the evolving branching process for a Poisson distribution**. *Top*) The probability of establishment (colors) was plotted as a function of the maximum replacement number and the initial replacement number. For the range of values plotted here, the probability of establishment ranged from 1 in 10 to 1 in a million. *Bottom*) The number of generations that elapsed before successful establishment, plotted for the same fitness values, ranged from 12 to 80.

There is always some chance that a parasite would fail to establish, but establishment was most unlikely when the fitness was initially lower than *1 *(Figure 3). The probability of establishment was also directly related to the maximum attainable fitness. As the maximum attainable fitness fell, so too did the probability of establishment. Reducing drug pressure would lower both the initial and maximum attainable fitness. When the initial fitness was *0.7 *(measured as relative to the sensitive parasite) and asymptotic fitness was *1.05*, approximately one mutant per million would establish (i.e., *Q ≈ 10 *^*-6*^). The analysis also suggested, not surprisingly, that more generations were required for establishment when either initial or asymptotic fitness was lower. In those parasites that did become established, the number of generations that elapsed prior to establishment ranged from 12 to 80, a delay of two to seven years.

### Spread

The spread of resistance was simulated using a simple epidemiological model that describes the dynamics of malaria. The models consider the fraction of humans in a population who are asymptomatically infected with drug-resistant or drug-sensitive parasites, *x *or *w*, respectively (Figure 1, bottom). The models also consider the incidence of clinical malaria in relation to infection status in a very general way (Additional file 1).

A therapy was considered to have "failed" when the frequency of resistance (i.e. *x/(x + w)*) exceeded a predefined threshold; the WHO threshold of 10% was adopted. The end of the branching process was the starting point for the epidemiological model, so in a population of *H *humans, the initial frequency of resistance was *100/H *humans. The time to reach a frequency of 10% was longer in larger human populations where the initial frequency was lower (Figure 1, bottom).

## Results and Discussion

### Reducing parasite fitness

**Figure 4:**
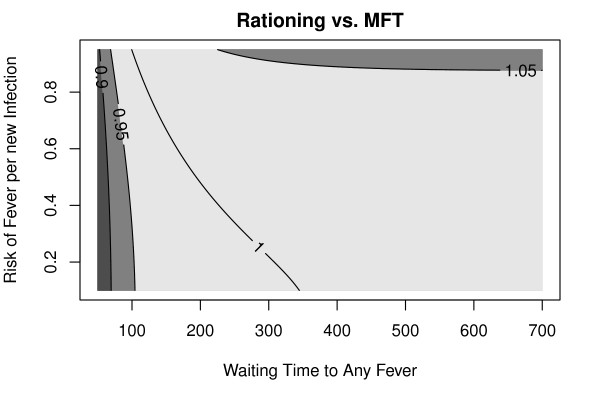
**A comparison of parasite fitness under strategies of rationing drugs to treat 50% of cases vs. two first-line ACTs**. This figure assumes that baseline drug pressure is 40% and that the biological cost is 10%. The waiting time to clinical malaria (i.e. any fever) in those with infection (x-axis) and the proportion of new infections that present with clinical symptoms (y-axis) span the relevant values for malaria. When the profile for clinical malaria resembled low-transmission settings, the benefits of MFTs were slightly lower than treating half as many patients. When the profile resembled high-transmission settings, MFTs did slightly better than rationing drugs. The colors shade in different categorical descriptions of the ratio. For most values, the ratio is within 5% of 1 (light gray).

To illustrate how multiple first-line therapies would reduce resistant parasite fitness, the reductions in drug pressure achieved using an MFT policy was compared to a drug rationing policy. The fitness of resistant phenotypes in a population that uses two first-line therapies was similar to the fitness of resistant phenotypes with a single first-line therapy where half as many people were treated and cured (Figure 4). This was not surprising, since the point of an MFT is to treat half the patients with one drug while treating the other half of the population with a different drug.

### Reducing drug pressure and time to failure

The expected waiting time to drug failure, denoted *T_F_*, considers appearance, establishment, and spread:.

**Figure 5:**
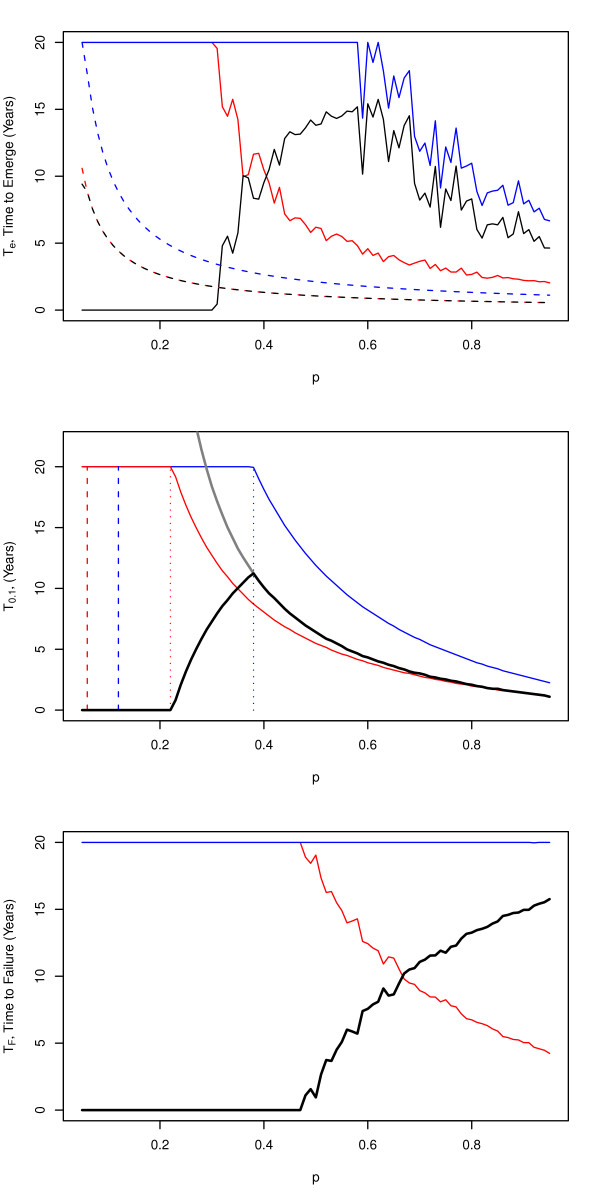
**Reducing drug pressure will delay emergence and slow spread**. The benefits of halving drug pressure can be enormous, but they must be considered within a finite time horizon. The x-axis shows baseline drug pressure. Baseline waiting times are plotted in blue. Baseline waiting times for halving drug pressure are plotted in red. The net benefit (the difference) is plotted in black. *Top*) Time to emerge (solid lines) combines the time to appear (dashed lines) with stochastic establishment. *Middle*) Clinical efficacy would be preserved for 20 years if drug pressure were low (red). Reducing drug pressure (blue) has a benefit in an intermediate range--when drug pressure is not low enough to make an intervention necessary but not high enough that resistance will rapidly fix, even if reduced. *Bottom*) For a reference parameter set, the combined effects of delaying emergence and slowing spread guarantee that MFTs will last 20 years. For high baseline drug pressure, the estimated waiting time to failure was as low as 5 years.

Reducing drug pressure delays appearance, reduces the likelihood of establishment, and slows spread. The expected waiting time to appearance was exactly twice as long when drug pressure was cut in half (Figure 5, top). The waiting time to establishment was approximately twice as long, too, through a combination of delayed appearance, an increase in the number of generations required to establish, and an increased likelihood of stochastic fadeout.

Reducing drug pressure also slows the spread of resistance. The benefits of extending a policy are complicated because of a threshold on drug pressure, a tipping point that favors drug-sensitive or drug-resistant parasites (Additional file 1). If reducing drug pressure takes selection from one side of the tipping point to the other, then it would delay drug failure indefinitely (Figure 5b). The quantitative benefits of reducing drug pressure, however, are much lower if selection stays on the same side of the tipping point. If drug pressure were low enough that drug-sensitive parasites were favored, there would be no need to reduce drug pressure further. If drug pressure were sufficiently high that it favored drug-resistant parasites before and after adopting a policy, reducing drug pressure would approximately double the time to failure (Figure 5b).

Decisions to preserve the efficacy of anti-malarial drugs are often made over a finite time horizon; here, the goal was preserving drug efficacy over 20 years. The introduction of a finite time horizon for planning is similar to the analysis of tipping points. If the goal of a policy is to preserve a drug for at least 20 years, then the analysis must reduce drug pressure low enough to achieve this goal. If the waiting time to failure for a single ACT were longer than 20 years, then there would be no need to do anything. A maximum benefit of deploying MFTs is found when a single ACT would delay resistance by about a decade. The plot of years gained up to a maximum of 20 years plotted against drug pressure, has the shape of a shark fin (Figure 5b). The combined effects of delayed emergence and delayed spread all combine to extend the useful therapeutic life of a drug (Figure 5c).

The most important principle is that the benefits of implementing a policy in terms of a waiting time to failure are all proportional to the waiting time to failure in the absence of any policy. It follows that a policy to preserve a drug is worth implementing only if the waiting time to fail is in the right range; if the waiting time to fail is less than one year, then a policy must extend the useful life of a drug by a factor of 20 or more, a practical impossibility. If the drug already has a waiting time of more than 20 years, then a policy is not really necessary. This principle transcends the uncertainty and makes it possible to say something more concrete about which drugs are worth protecting with a policy.

### Uncertainty and scale

The time to failure depends on the drug pressure, the mutation rates, the biological cost of resistance over time, the clinical incidence, and the generation time of the parasites. There is substantial uncertainty about all these parameters. To weigh the value of deploying MFTs, a probability distribution function was assigned to the parameters to compute times to failure (Additional file 1). The answers were generally uninformative.

Because there was so much uncertainty in all of the parameters, a slightly different question was asked: when would MFTs be most likely to provide a benefit? Asking the question in this way and doing a sensitivity analysis showed that the scale of the question was a dominant factor in the analysis; the more people with parasites who were treated, the shorter the time to appearance, but the longer the waiting time to reach a frequency of 10% because of a smaller initial frequency of resistance. The question of the appropriate scale came to dominate the analysis when it was noted that reducing the scale could increase the waiting time to appearance by millennia, depending on the size of the population at risk. Given the potential for ACT resistance to emerge anywhere and spread to nearly everywhere, as was the case with resistance to chloroquine and sulfadoxine-pyrimethamine [21], the global scale was considered to be appropriate.

Parasite population density at the time of treatment can vary by several orders of magnitude, which has a strong effect on the probability of appearance. Clinical malaria in an immunologically naïve adult can produce parasite densities that range up to *10 *^*12 *^parasites, in extreme cases. In areas with highly endemic malaria and clinical immunity, adults tend to have less clinical malaria with lower parasite densities at the time of treatment. By comparison, children are often immunologically naïve, but they have less blood volume and a smaller number of parasites. Reasonably large parasite population sizes were considered (assuming *10 *^*9 *^*< P < 10 *^*12*^).

The probability of appearance in an individual is extremely low, thus the waiting time to emergence somewhere in the world must account for the large populations at risk and global demand for drugs. There are approximately 450 million clinical malaria episodes per year, with a credible range of 350-550 million cases [22]. If ACT were scaled up to meet global demand, there could be as many as 5 billion courses of ACT per decade. For a single combination therapy to delay the appearance of resistance by a decade, assuming that 1 billion people were treated over a decade, the probability a resistant mutant appears per treatment would have to be less than approximately *10 *^*-18*^.

Mutation rates in the dihydrofolate reductase gene of *P. falciparum *are estimated to be approximately 2.5 × 10^-9 ^[23]. If ACT resistance required two such mutations of similar frequency, the mutation rate required for resistance to an ACT would be closer to *10 *^*-17*^. By this arithmetic, the waiting time to appearance somewhere in the world is close to a year. For a reference set of parameters (Additional file 1), the estimate of the waiting time to failure, after scaling up ACT, was approximately eight years. There was, it should be recalled, very little confidence in this particular estimate.

One notable feature of this analysis was that it combined very small numbers (mutation rates) with very large numbers (parasite population densities, the global demand for anti-malarial drugs). A small change in one of the exponents could change the time to failure by a factor of 10 or more. The proportional changes that would be achieved by implementing MFTs were most important for policy when the waiting times were large enough to have a noticeable effect but not already sufficient to preserve ACTs for 20 years. Curiously, when the big numbers were combined, they roughly balanced, and the waiting time to appearance, at a point in time when ACTs are the *de facto *global first-line treatment for malaria, would be close to one year, precisely the value where MFTs would have maximum benefit. As an example, MFTs delayed the waiting time to failure from 8 to 17 years (Figure 2).

## Conclusions

Treating fewer patients would likely reduce selection pressure and slow the spread of resistance, but there are few practical ways of reducing drug pressure without putting lives at risk. MFTs are one way: treating half the population with one ACT and the other half with a different ACT would reduce the fitness of resistant parasites approximately as well as treating half as many patients [8]. A simple rule of thumb for deciding whether to choose MFTs is that if there is an advantage to cutting drug pressure by half or more, then there is an advantage to deploying two or more ACTs.

This analysis suggests that MFTs are deployed to best effect on different segments of the same population. One possibility would be to distribute one ACT for home-based care and use a different ACT in the clinic. Another promising strategy would partition the market by age: paediatric patients would be given one ACT, adults another. The paediatric and adult markets are already partitioned because adults and children require different formulations. Implementing MFTs would introduce operational challenges, but choosing different first-line ACT for paediatric and adult populations would work as a solution within a set of operational challenges that already exist.

MFTs are deployed to best effect before resistance emerges, but such decisions must be made in the face of massive uncertainty. Despite uncertainty, a strategic policy decision to protect artemisinin by promoting universal use of ACT has been proposed and partially implemented [24]. If mutation rates for artemisinin resistance were known, the waiting time to the global emergence of ACT resistance could be calculated with increased confidence, and it would then be possible to evaluate whether existing policies were adequate. Recently, stable artemisinin resistance has been demonstrated in a murine model [25] and artemisinin-resistant infections were described along the Thai-Cambodia border [26] where artemisinin-class drugs have been used in Southeast Asia for twenty years [27]. While, the rate that artemisinin resistance spontaneously appears is apparently low, its emergence as usage rates dramatically increased raises the question of if it is low enough to preserve ACT until a new class of anti-malarial drugs is available? The answer will not be known with confidence until after ACT resistance has fully emerged. Meanwhile, uncertainty will remain high.

Surprisingly, this analysis arrived at a robust conclusion despite the epidemiological and biological uncertainties. In small and isolated populations, combination therapies could have a huge benefit by delaying the time to appearance, so the benefit of MFTs would be comparatively small. In the global human population at risk of malaria, the enormous benefits of combination therapies shrank because ACT resistance could emerge anywhere and spread everywhere, and the relative benefits of delaying establishment and spread using MFTs were comparatively large.

Our study suggested that, with wide confidence intervals, the waiting time to the global emergence of resistance to ACT was approximately a decade. MFTs would provide some insurance against this calamity by delaying emergence further and then slowing the spread of resistance. The same logic would support a triple combination--three drugs given simultaneously, resistance to which requires three independent mutations. A triple combination would almost certainly delay the appearance of resistance by a decade or more if the genetic basis for resistance to all three components were mutually independent, but no such therapy is available today.

In retrospect, the results reflect that ACT resistance is unlikely to appear in any small population, but it will appear with virtual certainty in a large one, thus slowing spread has a comparatively large benefit at the global scale. Variability in drug pressure among populations would likely depend on access to health care, the level of clinical immunity, and socioeconomic status. The populations of the world can be considered a mosaic of sources and sinks for resistance, depending on whether resistance is favoured or not. In such a landscape, MFTs will shift the balance and increase the proportion of sinks that would naturally contain ACT resistance. Combination therapies are designed to make appearance a truly rare event, but if they don't work as well as intended, MFT strategies provide an insurance policy by delaying emergence further and by limiting spread after emergence. MFTs are thus a natural complement to combination therapies.

Combination therapies for malaria were adopted in the face of uncertainty. The basis for deploying artemisinin combination therapies comes mainly from *a priori *arguments about mutation rates and from experience with other drugs, such as the success of the combination drug sulphadoxine-pyrimethamine over pyrimethamine alone. The case for MFTs must be made on the same basis as the case for combination therapies but with a greater degree of uncertainty. Since the loss of artemisinin would likely result in the loss of all ACTs, existing ACTs must be used in the best possible way. Despite the uncertainty, the analysis makes a strong *a priori *case for a coordinated global policy, like the global subsidy for ACTs, to promote heterogeneous drug policies to ensure the long-term therapeutic efficacy of ACTs.

## List of abbreviations

Artemisinin combination therapy (ACT); Multiple first-line therapies (MFTs)

## Competing interests

The authors declare that they have no competing interests.

## Authors' contributions

DLS & RL designed the study. DLS developed the models, produced the graphs, and wrote the first draft. DLS, RL, EYK, and FEM all contributed to revising and editing the manuscript and contributed to its final form and content. All authors approved the final version of the manuscript.

## Supplementary Material

Additional file 1 **Methods for Estimating Appearance, Emergence, and Spread**. A longer and more detailed description of the methods. Click here for file
