# Supplementary material for: Prospective strategies to delay the evolution of anti-malarial drug resistance: weighing the uncertainty
Source: Malar J. 2010 Jul 23;9:217. doi: 10.1186/1475-2875-9-217 (PMC2916917; doi:10.1186/1475-2875-9-217)
Supplement: Additional file 1 — Methods for Estimating Appearance, Emergence, and Spread. A longer and more detailed description of the methods. [file 1475-2875-9-217-S1.PDF]

Methods for Estimating Appearance, Emergence, and Spread

David L. Smith, Eili Y. Klein,
F. Ellis McKenzie, Ramanan Laxminarayan

Time to Appearance

The waiting time to appearance is computed in the following way:

1. Compute the probability of appearance in a person who is treated: $A_{P,m} = mP$ or if $A_{P,m} >$ 0.01, then $A_{P,m} = 1 - (1 - m)^P$. The default mutation rate was set to $m = 10^{-17.2}$, and the default parasite population density was set to $P = 10^{10.5}$.

2. Compute the waiting time for some distribution Y(P). Here, we assumed that Y(P) was approximately log-normal. The default variance of this distribution was set to 0.3 (on a log scale).

3. The probability of appearance, per treated person, would be $Z = \int_0^\infty Y(P) A_{P,m} dP$.

4. The expected number of appearances after treating pLH individuals would be $pLHZ$ (i.e. when L is the rate of clinical incidence and the expectation is taken over one unit of time), and assuming that the treatment rate is approximately constant, the waiting time to appearance is exponentially distributed with mean $(pLHZ)^{-1}$. We set a default value on pLH of 10^7.

For the default values, there were two appearances per year.

Time to Emergence

Stochastic persistence of *de novo* mutants was modeled as an evolving branching process, where the replacement number of a resistant phenotype was denoted $R_x(p,0)$ when it first evolved, with a maximum possible value of $R_x(p,\infty)$. This larger value was called the asymptotic fitness. In simulations, $R_x(p,\infty)$ was given by the replacement number that was computed by the simple epidemiological models.

We assumed that the number of offspring for each individual was Negative Binomial with mean $R_x(p,t)$ and variance κ, and the offspring would inherit their parent's fitness, with some mutation $R_x(p,t+1) = R_x(p,t) + \varepsilon$. The branching process "fails" when all the offspring have gone extinct, and "succeeds" when there are enough mutants with replacement numbers greater than one such that the risk of extinction was reasonably low. At this point, the spread of resistance is considered in the deterministic epidemiological model. The time to emergence was estimated from the average number of failures from 1000 trials before having one success.

To model subsequent mutations and the accumulation of compensatory mutations, we focused on changes in the fitness relative to the asymptotic fitness. The size of the mutation was centered at zero, with a distribution given by: $\varepsilon(\sigma, \beta, R_x(p,t), R_x(p,\infty)) = [R_x(p,t) - R_x(p,\infty)]\, B(\alpha,\beta) - \sigma$, where $\alpha = \sigma\beta / (1 - \sigma)$ using the default parameterization for the *beta* distribution, B, that comes in the computer software package R.

The biological cost at any generation after appearance is defined as the fitness in absence of drug pressure, $R_x(p,t)$. By considering the accumulation of compensatory mutations in the evolving branching process after the appearance of resistance, we are explicitly assuming that there is a *de novo* biological cost, $R_x(0,0)$, that is higher than the asymptotic biological cost, $R_x(0,\infty)$. The quantities κ and drug pressure, p, and the probability distribution function of the mutations in fitness, ε, define the waiting time to emergence.

Epidemiological Model for Spread

A therapy was considered to have "failed" when the frequency of resistance exceeded a predefined threshold; we adopted the WHO threshold of 10%. We simulated the spread of resistance and computed the waiting time to failure using simple epidemiological models. The model also serves as a heuristic to define parasite fitness, the expected number of new hosts that become infected, in terms of drug pressure, p, and other epidemiological parameters. The epidemiological model considers the fraction of a human population that is uninfected (u), infected with sensitive (w), or resistant parasites (x). The frequency of resistance is $x / (x + w)$.

In the models, some aspects of fitness are related to the within-host biology of the parasites, and other aspects are determined by the epidemiology and by drug pressure. The need for drugs is defined by the fraction of new infections that present with clinical symptoms, ξ, and the rate of all-cause fever in those with parasites, Y. As a link to the first model, clinical incidence would be $L = (V\xi u + Y(w+x))H$. We have assumed that transmission rates are proportional to vectorial capacity, V, and the proportion of the population that is infected. The waiting time to clear an untreated infection is $1 / r_w$ or $1 / r_x$, for sensitive and resistant parasites, respectively. The dynamics of resistance are described by the equations:

$$\begin{aligned} \dot{w}/w &= Vu(1-\xi p) - r_w - p\psi \\ \dot{x}/x &= Vu - r_x \end{aligned} \qquad (1)$$

The replacement numbers for the parasite phenotypes in the absence of drug treatment are $R_{0,w} = V / r_w$ and $R_{0,x} = V / r_x$. The presence of drugs lowers the reproductive output of the drug-sensitive parasites to $R_w(p) = V(1 - \xi p) / (r_w + pY)$.

The potential for resistant phenotypes to invade a population is defined in an environment where malaria is endemic and resistance is absent. Before resistance has emerged, the frequency of the drug-sensitive type in response to selection pressure is $\overline{w} = 1 - 1/R_w(p)$. The expression for the fitness of the resistant types in this environment comes from criterion for invasion:

$$\dot{x}/x\big|_{\overline{w}} = V/R_w(p) - r_x > 0 \qquad (2)$$

or equivalently:

$$R_x(p)/R_w(p) > 1. \qquad (3)$$

The biological cost of resistance is defined by: $[R_w(0) - R_x(0)] / R_w(0) = 1 - r_w / r_x$.

The model can be extended to consider two drugs or therapies, with corresponding drug pressures p_x and p_y. We consider the dynamics of the phenotype that is resistant to each drug or therapy and to both. The fraction colonized by parasites resistant to each type is x and y, and the fraction colonized by both drug-resistant types is z. For convenience, we let $u = 1 - w - x - y - z$ denote the fraction that is not infected:

$$
\begin{aligned}
\dot{w}/w &= V\left(1 - \xi\left(p_x + p_y\right)\right)u - r_w - \left(p_x + p_y\right)\psi \\
\dot{x}/x &= V\left(1 - \xi p_y\right)u - r_x - p_y\psi \\
\dot{y}/y &= V\left(1 - \xi p_x\right)u - r_y - p_x\psi \\
\dot{z}/z &= Vu - r_z
\end{aligned}
\qquad (4)
$$

The basic reproductive numbers are $R_{0,w} = V / r_w$, $R_{0,x} = V / r_x$, $R_{0,y} = V / r_y$ and $R_{0,z} = V / r_z$. We assume that $r_z > r_x = r_y > r_w$, so the biological cost of the multi-drug or multi-therapy resistant mutant is higher than for resistance to just one drug or therapy.

In the presence of drugs, the replacement numbers are:

$$
\begin{aligned}
R_w\left(p_x + p_y\right) &= \frac{V\left(1 - \xi\left(p_x + p_y\right)\right)}{r_w + \psi\left(p_x + p_y\right)} \\
R_x\left(p_y\right) &= \frac{V\left(1 - \xi p_y\right)}{r_x + \psi p_y} \\
R_y\left(p_x\right) &= \frac{V\left(1 - \xi p_x\right)}{r_y + \psi p_x}
\end{aligned}
\qquad (5)
$$

Before resistance has emerged, the frequency of the drug-sensitive type in response to selection pressure is $\overline{w} = 1 - 1/R_w(p_x + p_y)$. The expression for the fitness of the resistant types in this environment comes from the criterion for invasion:

$$\dot{x}/x\big|_{\overline{w}} = V(1 - \xi p_y)/R_w(p_x, p_y) - r_x - \psi p_y > 0. \qquad (6)$$

A similar expression describes invasion of resistance to the other drug. This can be rearranged to:

$$R_x(p_y)/R_w(p_x, p_y) > 1. \qquad (7)$$

If each first-line therapy were deployed in equal amounts, with the same total fraction of clinical malaria cases treated (p), then the replacement number of a parasite that is resistant to one focal therapy is:

$$R_x(p) = \frac{1 - \xi p/2}{r_x + \psi p/2} \cdot \frac{r_w + \psi p}{1 - \xi p}. \tag{8}$$

The replacement number of the multi-drug resistant type is:

$$R_z(p) = \frac{r_w + \psi p}{r_z(1 - \xi p)}. \tag{9}$$

We assume that multi-drug resistance does not already exist and that it does not become a concern before the first ACT fails.

Time to Failure

To compute a waiting time to drug failure for any set of parameters, we followed the following algorithm:

1. Compute the waiting time to appearance to a single, focal drug or therapy: $T_a = p_x \mathrm{L} H m_x \int P\, \Psi(P)\, dP$

2. Compute the maximum replacement number $R_x(p_x, p_y)$, the biological cost of resistance $1 - r_x / r_w$, and the initial fitness $R_x(p_x, p_y, 0)$.

3. Compute the average probability of establishing and the mean number of generations elapsed, Q and G.

4. The time to emergence was $T_e = T_a / Q + G T_g$.

5. Compute $T_{10\%}$, the waiting time to reach 10% from an initial frequency of $100 / H$ by solving the two drug equations.

6. The waiting time to failure is the waiting time to emerge and then spread; for any set of parameters, this can be computed as: $T_e + T_{10\%}$.

Parameters

p - The fraction of clinical episodes that are appropriately treated probably varies from a high of nearly 100% in places with excellent health to a low of nearly no treatment in remote places with poor access to healthcare infrastructure.

m – The spontaneous mutation rate per cell division

H – The size of the human population at risk of malaria is approximately 0.98 billion in areas of very low or unstable risk, and approximately 1.47 billion people who live in areas of stable risk.

L*H* – The number of clinical episodes of *P. falciparum* malaria has been estimated at 515 million.

Y(*P*) – Parasite population densities at the time of treatment are probably maximal in adults who lack immunity; extremely high densities range up to 10^{12}. Across the world, parasite densities are lower in children who have less blood and in people who have developed anti-parasite immunity. We take a mean log-density of $P \approx 10^{10.5}$ with a variance in the log parasite density of 0.6.

$R_x(0)$ / $R_x(\infty)$ – There is little information on the initial biological cost, compared with the asymptotic biological cost, but we consider that a higher initial cost is plausible, for many reasons. We consider costs that range up to 30%.

ε – The size of a mutation was a scaled *Beta* function with mean 0. The default values that we used were $\sigma = 0.03$ and $\beta = 100$.

κ – The number of offspring in the branching process was drawn from a negative binomial distribution with aggregation parameter κ. For $\kappa = 0$, the number of offspring is drawn from a Poisson.

T_g – The generation time was taken to be approximately two months. The shortest time to complete a single generation is approximately one month. It includes the 10-day incubation period in humans, the 8 day waiting time for gametocyte maturation, and the 10-12 day waiting time for sporogony in the mosquito. The duration of the infectious period in humans is approximately six months.

ξ – The proportion of new infections in uninfected hosts that result in clinical malaria. In areas with no immunity, $\xi \approx 1$. In areas with some immunity, the values in adults and children will differ. We considered a broad range.

Y – Fever from all causes, including malaria, can arise in people who are already infected. The waiting time for a febrile event varies by age and malaria immunity. Here, we considered a broad range of values.

r_w – The time to clear an untreated, simple infection is approximately 200 days [1].

$1 - r_w / r_x$ – The biological cost of resistance was assumed to range between 0 and 30%.

R_0 – The values of R_0 were between 1.5 and 100. Vectorial capacity was used for the simulations; it was computed from the formula $R_0 = bcV / r_w$, where $bc \approx 0.4$.

We conducted extensive uncertainty analysis (not shown), but finally concluded that trying to make any conclusions based on total uncertainty would be meaningless. For any particular parameter set, the time to emergence could from one day up to more than a century. In our experience, total uncertainty only served to confuse the issues. Each one of these parameters was an important source of uncertainty. So was the model itself. However complicated the assumptions in other parts of the model, the biggest sources of uncertainty were: 1) the mutation rates, which could vary by several orders of magnitude; 2) The initial and maximum replacement numbers; and 3) the frequency of compensatory mutations. If the maximum replacement number of the drug-resistant parasite was less than that of the drug-sensitive parasites, resistance would never invade. If the initial replacement number was less than one, the probability of establishing could plausibly be very low–on the order of requiring an extra mutation. Thus, although there might be some marginal value to refining the models, the same sources of uncertainty would still dominate the analysis.

Table 1: The parameters in the model. Parameter values are justified in the text

**Appearance**		
p	variable	The fraction of clinical episodes appropriately treated
m	$\approx 10^{-17.2}$	The spontaneous mutation rate, per cell division
H	2.5×10^{9}	The size of the human population at risk
LH	500 million	Global number of clinical episodes of malaria per year
P	see Y(P)	Parasite density at the time of treatment
Y(P)	lognorm(10.5, 0.6)	The probability distribution function of P
T_a		Time to appear
**Establishment**		
$R_x(p,\infty)$	varies with p	The maximum replacement number of a *de novo* mutant
$R_x(0,\infty) / R_x(p,\infty)$	unif(0.7,0.99)	The higher initial biological cost
ε	scaled *Beta*	The size of a mutation
κ	0	From negative binomial distribution (Poisson for $\kappa = 0$)
Q		The probability of establishing
G		The number of generations to establish
T_g	$\approx 60d$	The average parasite's generation time
T_e	$T_a / Q + GT_g$	Time to emergence
**Spread**		
p		(see above)
ξ	0.1 – 1	Clinical malaria in uninfected hosts, per infection
Y	30-700 d	Waiting time to clinical malaria in infected hosts
r_w	200 d^{-1}	Time to clear untreated infections, wild-type
$1 - r_w / r_x$	unif(0.7,0.99)	The biological cost of resistance
$R_w(0)$	1.5 – 100	Basic reproductive number for wild-type
u		The fraction uninfected
w		The fraction infected with the wild-type
x		The fraction infected with parasites resistant to therapy x
y		The fraction infected with parasites resistant to therapy y
$T_{10\%}$		The time to reach 10% resistant parasites
**Failure**		
T_F	$T_e + T_{10\%}$	Time to failure

References

1. Eyles DE, Young MD (1951) The duration of untreated or inadequately treated Plasmodium falciparum infections in the human host. Journal of the National Malaria Society 10: 327-336.
